# Travel with your kin ship! Insights from genetic sibship among settlers of a coral damselfish

**DOI:** 10.1002/ece3.6533

**Published:** 2020-07-14

**Authors:** Vanessa Robitzch, Pablo Saenz‐Agudelo, Michael L. Berumen

**Affiliations:** ^1^ Red Sea Research Center Division of Biological and Environmental Science and Engineering King Abdullah University of Science and Technology Thuwal Saudi Arabia; ^2^ Instituto de Ciencias Ambientales y Evolutivas Facultad de Ciencias Universidad Austral de Chile Valdivia Chile

**Keywords:** coral reefs, damselfishes, genetic sibship, larval dispersal, larval recruitment, population dynamics

## Abstract

Coral reef fish larvae are tiny, exceedingly numerous, and hard to track. They are also highly capable, equipped with swimming and sensory abilities that may influence their dispersal trajectories. Despite the importance of larval input to the dynamics of a population, we remain reliant on indirect insights to the processes influencing larval behavior and transport. Here, we used genetic data (300 independent single nucleotide polymorphisms) derived from a light trap sample of a single recruitment event of *Dascyllus abudafur* in the Red Sea (*N* = 168 settlers). We analyzed the genetic composition of the larvae and assessed whether kinship among these was significantly different from random as evidence for cohesive dispersal during the larval phase. We used Monte Carlo simulations of similar‐sized recruitment cohorts to compare the expected kinship composition relative to our empirical data. The high number of siblings within the empirical cohort strongly suggests cohesive dispersal among larvae. This work highlights the utility of kinship analysis as a means of inferring dynamics during the pelagic larval phase.

## Graphical abstract

Using Monte Carlo simulations and next generation sequencing, we found strong evidence of significantly high presence of siblings within a recruiting cohort of a coral dwelling damselfish, in the Red Sea. To our knowledge our study is the first to assess the significance of kinship among new settlers thus, we provide a pipeline and computational tool to encourage similar future studies. Such information is substantial for the management of marine populations.

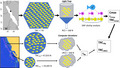

## 1 INTRODUCTION

Most coral reef fishes have a bipartite life cycle with a benthic adult stage, during which movement between reefs is highly restricted, and a pelagic larval phase, which presents the primary opportunity for dispersal. After completion of the pelagic larval duration (PLD), the larvae return to reefs to settle and are referred to as new recruits. From the moment fish larvae hatch to the day they recruit, they are exposed to a wide range of environmental factors that determine their fate and final location of settlement, undergoing the highest mortality rates of their lifetime, which among coral reef fishes may further increase as they approach intense predation pressure (the so‐called “wall of mouths”) at the reef of settlement. The PLD can last from several days to months, depending on the species, which allows for presumed interspecific differences in dispersal potential and local genetic population structures (Eble, Toonen, & Bowen, 2009; Selkoe & Toonen, 2011; Selkoe et al., 2010; Weersing & Toonen, 2009). Larvae of coral reef fishes were once considered passive particle‐like objects with little to no significant swimming behavior and therefore were assumed to be transported solely by oceanographic processes until they reached a reef for settlement (Leis, 1991; Roughgarden, Iwasa, & Baxter, 1985; Warner & Hughes, 1988). We are now aware that the PLD is much more complex and unpredictable than previously thought, because many of these larvae can have extraordinary navigation skills and may respond to sensory cues (Gerlach, Atema, Kingsford, Black, & Miller‐Sims, 2007; Lecchini & Nakamura, 2013), have strong and efficient vertical and horizontal swimming behavior even against currents (Leis & Mccormick, 2002), perform orientated navigation (following, e.g., sunlight (Mouritsen, Atema, Kingsford, & Gerlach, 2013)), and possibly intentionally travel and/or settle with their kin (Shapiro, 1983; Buston, Fauvelot, Wong, & Planes, 2009; Madduppa, Timm, & Kochzius, 2014; but see Avise & Shapiro, 1986). Thus, larval behavior has increasingly gained attention (reviewed in Kingsford et al., 2002; Leis, 2006; Montgomery, Tolimieri, & Haine, 2001) as it has ramifications for the maintenance and dynamics of marine populations (Paris & Cowen, 2004; Werner et al., 1993). However, difficulties associated with tracking tiny larvae (Staaterman & Paris, 2014) in the vastness of oceans have hampered the study of such early life stages.

Studies on the dispersal strategies of early life histories of coral reef fishes have focused on later development stages using genetic kinship analyses and/or abundance surveys of settled juveniles (Almany et al., 2013; Avise & Shapiro, 1986; Bernardi, Beldade, Holbrook, & Schmitt, 2012; Berumen et al., 2012; Herrera et al., 2016; Jones, Planes, & Thorrold, 2005; Nanninga, Saenz‐Agudelo, Zhan, Hoteit, & Berumen, 2015; Schunter, Garza, Macpherson, & Pascual, 2013). Interestingly, most of these studies found evidence for the presence of siblings and high genetic relatedness between some of the juveniles studied. For instance, Bernardi et al. (2012) were the first to detect that siblings of a damselfish were arriving together at their new home, as they found that related individuals mostly recruited to the same or nearby anemones on the same night (Bernardi et al., 2012). However, whether this was just an anomalous result due to high recruitment success of a small recruiting source to that particular reef or for that species, or if it was evidence for some intrinsic behavioral trait of a fish larva to stay close to its kin during the PLD, still remains unanswered.

Our study focuses on *Dascyllus abudafur*, a small zooplanktivorous coral reef damselfish, which was recently taxonomically “resurrected” from its sister species, *D. aruanus* (Borsa, Sembiring, Fauvelot, & Chen, 2014). These fishes form colonies in which all individuals differ in their total body length, likely indicating that they are of different age. Nonoverlap of ages may serve as a mechanism to decrease foraging competition and inbreeding among putative siblings of the same recruiting cohort (among *D. aruanus* (Gillespie, 2009) and in *D. abudafur*, from personal measurements). After the completion of a PLD of 23.09 (±1.92 *SD*) days (Robitzch, Lozano‐Cortés, Kandler, Salas, & Berumen, 2015) they reside in branching corals for their entire life span (among *D. aruanus* from Fricke & Holzberg, 1974; and Coates, 1982). *Dascyllus abudafur* is omnipresent in the Red Sea and the Western Indian Ocean (Borsa et al., 2014) while *D. aruanus* is abundant and well‐studied throughout the Indo‐Pacific. Therefore, ample information is available on *D. aruanus’* biology and ecology, which we use as proxy for the biological data implemented in our study for statistical inferences (Appendix S1). For *D. abudafur*, there is no information available on recruitment behavior. However, *D. aruanus* tends to have seasonal breeding peaks and preferentially recruits a couple of days after the new and full moon and around the first and third quarter moons, a pattern that is common among damselfishes (Mizushima, Nakashima, & Kuwamura, 2000). In our study, we assessed the genetic composition and relatedness of a subset of 168 new recruits captured in one single night during the new to first quarter moon, in one single reef in the north central Saudi Arabian Red Sea, which we refer to as the recruiting cohort sample (RCS). This RCS provides a snapshot of the geneflow for a specific reef during one recruitment event and allowed us to ask crucial questions about larval behavior at very early life stages. We were also provided some insight on realized connectivity between populations (Watson et al., 2010). Since it is nearly impossible to follow and observe a larva from the time it hatches to the time it settles, inference derived from sibship among the larvae of our RCS allows us to propose strategies of dispersal and putative cohesive routing during the PLD of this species. The main questions of this study were: (a) Are larvae nonrandomly recruiting together with their kin? and (b) does the RCS reflect the genetic diversity of the adult population at the settlement destination?

## 2 METHODS

### 2.1 Study site and sample collection of Dascyllus abudafur

**FIGURE 1:**
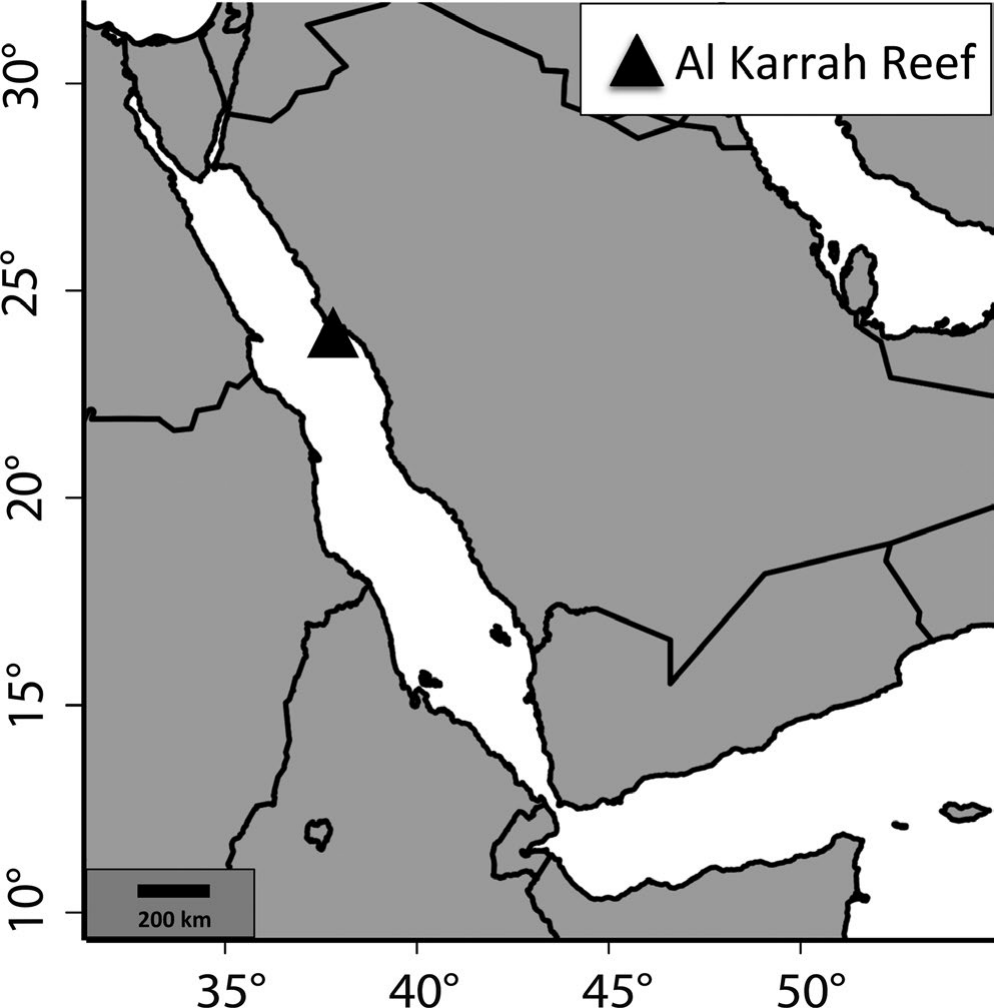
The sampling site of *Dascyllus abudafur* recruits in the Saudi Arabian north central Red Sea, Al‐Karrah Reef (N22°56.265; E38°45.943), indicated by a black triangle and located near the coastal city of Rabigh

In the Saudi Arabian Red Sea near the city of Rabigh at Al‐Karrah Reef (AKA; N22°56.265, E38°45.943; Figure 1), 171 *Dascyllus abudafur* recruits were collected around the first quarter moon on March 5th 2014, using a LED (12 V) powered cylindrical light trap (1 m long and 0.5 m diameter of 500 μm Nitex mesh netting; Bellamare). The light trap was deployed with a 1 kg weight attached to its cod end, from a 35 m research vessel (RV Thuwal) on the side of the reef sheltered from the predominant wave activity. The light trap remained at a depth of ~1 m and a bottom depth of ~15 m from 17.00 hr (before sunset) until 06.00 hr (before sunrise) of the next day. This sample is referred to as the recruiting cohort sample (RCS). Additionally, 53 adult specimens (referred to as the adult population sample; APS) were collected the same day at about 15 m depth from four coral colonies at AKA using clove oil, tweezers, hand nets, and a 1 m × 1.5 m plastic bag to cover the targeted colonies (before applying clove oil). All specimens were immediately stored in 96% ethanol and transported to KAUST facilities for further analysis.

### 2.2 Single nucleotide polymorphisms (SNPs) library preparation and sequencing

Genomic DNA was extracted from preserved fin or gill tissue (from adult specimens) or the head of the recruiting larvae using a Nucleospin‐96 Tissue Kit (Macherey‐Nagel, Düren, Germany). Double digest restriction associated DNA (ddRAD) libraries were prepared using the restriction enzymes *SphI* and *MluCI* (NEB) and following the protocol described by Peterson, Weber, Kay, Fisher, and Hoekstra (2012)(Peterson et al., 2012) with some modifications (see Appendix S2). In total, two libraries were created on a HiSeq 2000 Illumina sequencer, each containing 112 individuals providing single end reads (1 × 101 bp; v3 reagents).

### 2.3 De novo assembly and filtering

Sequences of 224 individuals were demultiplexed and quality filtered using the ‘process_radtags.pl’ pipeline in STACKS version 1.42 (Catchen, Amores, Hohenlohe, Cresko, & Postlethwait, 2011). Individual reads with phred‐scores below 30 (averaged on a sliding window of 10%) or with ambiguous barcodes were discarded. After this, 13 individuals with less than 500,000 reads were discarded. For the remaining 211 specimens, RADSeq loci were assembled de novo using the ‘denovo_map.pl’ pipeline in STACKS. Different parameter combinations were evaluated, which resulted in different numbers of loci but gave similar results in genetic comparisons (genetic clustering and *F*_ST_ between sites). For the main analyses presented herein, we used a parameter combination similar to the one recommended by Mastretta‐Yanes et al. (2014). Further data filtering was performed using the *population* component of STACKS (details in Appendix S3). The resulting vcf file was converted to other program‐specific input files using PGDSPIDER v2.0.5.2 (Lischer & Excoffier, 2012). The data set was evaluated for linkage disequilibrium (LD) and Hardy–Weinberg equilibrium (HWE), as implemented in Genepop 4.2 (Raymond & Rousset, 1995; Rousset, 2008), to choose a set of SNPs that were statistically independent to each other, met kinship analysis expectations, and was thus analytically highly powerful with a lower numbers of SNPs (for faster and accurate computational inferences). LD and HWE assessments were solely based on the genotypes of the 38 adult individuals (APS) to prevent false results of LD and HWE deviations in case of potentially higher relatedness or selective pressure among young recruits. P‐values were tested for false‐discovery‐rate using *qvalue* in R (package from BioLite). GenAlEx 6.501 (Peakall & Smouse, 2006, 2012) was used to calculate the multilocus probability identity, which provides an estimate of the average probability that two unrelated individuals drawn from the same population will have the same multilocus genotype. Using this information, 300 loci (max. probability identity of 7.11 × 10^–36^) with the most equally distributed allele frequencies (minor allele frequency > 0.33) were chosen as a highly informative and computational economic final data set, comprising 193 individuals with less than 15% missing data.

### 2.4 Population structure and sibship assignment

In order to identify potential genetic differentiation between the APS and the RCS, a Bayesian clustering analysis was performed in STRUCTURE v. 2.3.4 (Pritchard, Stephens, & Donnelly, 2000). The analyses were carried out with 300,000 iterations discarded as burn‐in and 500,000 iterations retained, averaged for ten runs for a number of populations set from *K* = 1 to 3. A maximum *K* of 3 was used to represent one step above the assumed putative maximum number of *K*’s (*K* = 2): one for the adult population and one for the recruiting cohort. CLUMPAK (Kopelman, Mayzel, Jakobsson, Rosenberg, & Mayrose, 2015) was used to choose and graph the optimal *K*. Additionally, a PCA was performed using the *dudi.pca* function in the R package *poppr* to see whether the two samples clustered separately or not depending on their genotype.

COLONY was used to identify sibling groups within the RCS. Sibling pair assignments were accepted upon a posterior probability exceeding 0.75 (as used for other coral reef fishes (Herrera et al., 2016; Schunter et al., 2013)). The parameters used were the following: polygamous diploid dioecious species, no inbreeding, unknown population allele frequencies, no information on the maternal or paternal mean sibship size, and using the FLPL method: a combination of full likelihood (FL) and the pairwise‐likelihood (PL) method, with medium precision in computation, faster than the FL method, but with a similar accuracy specially for high number of markers and not too large sibship sizes. These parameters were used on the 300 loci data set, using three different random seed numbers.

### 2.5 Estimation of the theoretical total recruiting cohort (TRC) of Dascyllus abudafur at Al‐Karrah Reef (AKA, Red Sea) for statistical inferences

**FIGURE 2:**
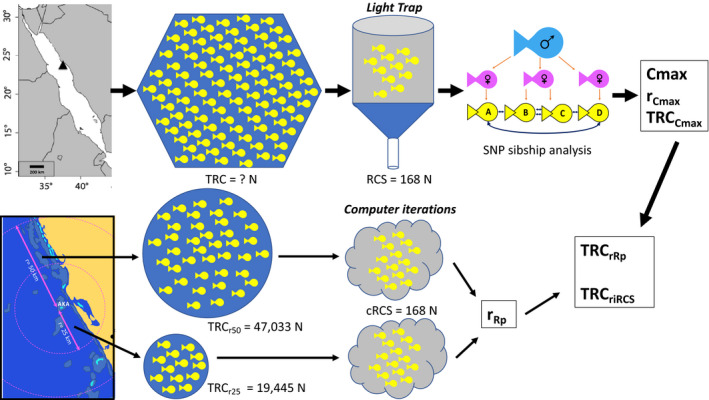
Scheme of the types data used in this study for *Dascyllus abudafur* from a north central Red Sea reef, Al Karrah (black triangle in the map, upper left corner). Yellow fish represent recruiting larvae of *D. abudafur*, for which “TRC” is the total recruiting cohort, represented by the different blue figures. The hexagonal blue figure is the TRC the study targets to estimate, while the two smaller round blue figures represent theoretical TRCs under the scenario that the radii for these TRCs are either 25 km (r25) or 50 km (r50). The gray figures represent a subsample of the recruiting cohort (i.e., recruiting cohort sample, “RCS,” either empirical or Monte Carlo simulated). The cylindrical gray figure symbolizes the light trap which captured the recruiting larvae in this study (i.e., our RCS of *N* = 168), for which ddRAD sequencing SNPs data was generated to estimate sibship assignments using the program COLONY (i.e., blue/male, pink/female, and yellow/progeny fishes). Based on these results, the maximum number of colonies that provided our RCS at AKA (Cmax), the theoretical radius around AKA containing Cmax (r_Cmax_), and the resulting TRC for that Cmax (TRC_Cmax_) were calculated. Additionally, computational subsampling of the two theoretical TRCs (for r25 and r50) provided computed RCSs (“cRCS,” *N* = 168), with which the maximum source radius for a putative TRC containing sibship assignments similar to those within our empirical RCS (r_Rp_) was estimated to jointly infer: the TRC from the maximum possible radius to achieve sibship assignments similar to those within our empirical RCS by random (TRC_rRp_) and the TRC of the maximum number of colonies that can be source of the RCS in order to achieve with 95% confidence a similar sibship assignment to that found within the empirical RCS (TRC_riRCS_). For more details see Table 2

To investigate whether the percentage of sibling pairs (calculated by COLONY) within our empirical RCS at AKA (*N* = 168, after quality‐filtering of SNPs data) were evidence of corecruitment with kin in *D. abudafur*, or whether similar values could also be achieved by random association, we estimated different putative theoretical total recruiting cohorts (TRCs). We define the TRC as the total number of recruiting larvae that were theoretically arriving at AKA on the night we took our sample of 168 recruits (the empirical RCS) with a light trap. The inferred TRCs were used to subsample, randomly and computationally, multiple recruiting cohort samples (computed RCSs, i.e., cRCSs) of equal size (*N* = 168) to our empirical RCS; and compare the sibship composition of both (the cRCS versus. our empirical RCS). Hence, we tested whether the sibship assignments within the empirical RCS were significantly higher than the average sibship composition of the permutated random cRCSs. A scheme of our experimental design can be found in Figure 2.

As a first step to estimate the size of and kin within a theoretical TRC, data on the reproductive biology, ecology, abundance, and habitat of *D. abudafur* was gathered from literature, personal observations, and discussions with expert colleagues in the field (Appendix S1 and Table 2). One problem with assessing whether siblings are more common than expected is that we do not know the area from which the recruiting cohort could have been sourced. Since the size and genetic composition of the true TRC is unknown, to have a wider insight into what kind of cRCSs different TRCs could deliver, the aforementioned information was firstly used to infer two theoretical TRCs of different source areas (sizes/radii) around our study site. The first one, “TRC_r25,_” represented the scenario that larvae hatching from reefs around a radius of 25 km (r25) from AKA can recruit to AKA after completing their pelagic larval duration (PLD). The second, “TRC_r50,_” represented the scenario that the AKA recruits have hatched at reefs from a radius of maximum 50 km (r50) around AKA. We think that these two radii fairly represent reasonable expectations for damselfish dispersal distances (Almany et al., 2017; Pinsky, Palumbi, Andréfouët, & Purkis, 2012).

For the calculations of the two TRC_r_s (TRC_r25_ and TRC_r50_), the following parameters were required: (a) the daily number of larvae produced by an average colony of *D. abudafur* that will survive the PLD and successfully recruit to a new home reef (i.e., be part of the daily TRC of a reef during the reproductive season; R_Cd_); (b) the number of *D. abudafur* colonies per area of habitat in the Red Sea (C_A_); (c) the percentage of simultaneously reproductive active females in the population (F_R%_); and (d) the area of habitat available in a chosen radius of (putative) recruitment sources (A_r_). This resulted in the following formula: TRC_r_ = R_Cd_ × C_A_ × F_R%_ × A_r_. The same formula was used for both radii since we did not incorporate a decay in dispersal with distance among *D. abudafur* in the Red Sea due to the lack of evidence for isolation by distance at a scale relevant to our study (Raitsos et al., 2017; Saenz‐Agudelo et al., 2015). The values for all formulae used can be found in Table 2, and details on the calculations of (a) to (d), in Appendix S4.

### 2.6 Statistical significance of kinship results: hypothesis testing

With the calculated TRC_r25_ and TRC_r50_, our study aimed to test the hypothesis (null hypothesis) that the presence of a given number of sibling pair assignments or sibling groups in our sample can be the result of random events rather than active larval behavior. Under this hypothesis, we assume that a pool of *N* recruits (here: *N* = 168) taken from the TRC at a certain reef is a random sample of a completely mixed recruitment cohort at the focal home reef. The alternative hypothesis is that those *N* recruits comprise groups of siblings because larvae stick together among their kin (and are not randomly mixed), while traveling in the pelagic environment (during their PLD), and before recruiting (as we believe is the case for our empirical RCS).

To test our hypothesis, a script was coded in PYTHON (https://github.com/HexTree/fish‐siblings/blob/master/fish_sim.py) for Monte Carlo simulations to randomly draw one million times a subsample of 168 recruits (= cRCSs) out of the theoretical TRC_r_s (TRC_r25_(1) and TRC_r50_(2)) and compare the average sibling composition of the random draws (average sibling composition of cRCS_r25_s vs. that of cRCS_r50_s) to calculate the rate (Rp) at which the likelihood to withdraw sibling pairs (SPs) in a cRCS (*N* = 168) decreases as the radius is halved. Rp was calculated using the following two assumptions: (e) 1⁄2 × r50 = r25 and (f) SPr25 = SPr50 × Rp. With the sibling pair assignments from the permutations (SPr25 and SPr50) and the obtained Rp (from (f)), we estimated how much smaller the radius of the TRC had to be in order to theoretically obtain a similar average sibling pair assignment to that calculated by COLONY in our empirical RCS, in that particular TRC (of that particular radius according to our previously defined Rp; i.e., TRC_rRp_(3)). The last TRC_r_, was calculated assuming the maximum number of colonies COLONY suggested to be the sources of our empirical RCS (Cmax), equal to the total number of colonies that produced the recruits that night at AKA (TRC_rCmax_(4); i.e., the TRC_r_ under the scenario that the empirical RCS = TRC).

Once TRC_r25_ and TRC_r50_ were calculated and used for permutations to obtain Rp and calculate our TRC_rRp_, TRC_rRp_ was tested for the frequency of occurrence of single recruits or: sibling pairs, triplets, and quadruplets (i.e., events of sibling pairs) within a random cRCS sample (of 168 individuals). Hence, the inferred TRC_rRp_ was subsampled (picking cRCSs of 168 individuals) 100,000 times and the results (of these random picks) explored for the events of sibling pairs. These observations were then used to (a) assign a total score value to each of the iterations depending on the frequency of each of the sibling pair events in each random pick of 168 individuals (i.e., each cRCS). The scores were indirectly proportionally assigned to the frequency of sibling pair assignments. The same scoring system was used (b) to assign a total score value to the empirical data set of 168 individuals (RCS) representative of the number of single recruits, sibling pairs, triplets, and quadruplets found by COLONY within our RCS. With this scoring system, (c) random samples were then simulated (cRCSs) and their score values computed to assess how frequent the score value of our data set was observed within the random cRCSs and the likelihood of the empirical results (i.e., how likely it was to find the assigned score value of the RCS among random picks (cRCSs) in the theoretical TRC_rRp_) as well as the significance behind the COLONY sibship assignments.

Finally, the same tool was used to infer the maximum theoretical radius of r_iRCS_ at AKA required to have a high likelihood of sampling cRCSs of a similar kinship to that of our empirical RCS solely by chance. In other words, r_iRCS_ would finally give us an estimate of how small the radius would have to be in order to be certain to achieve the empirical sibling pairs randomly and not due to putative larval behavioral preferences (e.g., cohesive dispersal with kin). To calculate this final r_iRCS_, the program inferred score values for different sized TRCs until the achieved score value of the permuted TRC was within a 95% confidence interval of the score value calculated for the empirical sample of TRC (i.e., when the score value of the cRCS sampled from TRC was within the 95% confidence interval of the empirical RCS’s score value); meaning that in that theoretical TRC, by picking 168 individuals (i.e., a random cRCS), there is a 95% chance of having a score value equal to the one assigned to our dataset/RCS. That TRC was called TRC_riRCP_ and was used to back‐calculate r_iRCS_ assuming six recruits per parental colony and 102 reproductive colonies per km^2^ of habitat (see C_A_, Table 2).

## 3 RESULTS

### 3.1 Raw sequence filtering and assembly

A total of 594,692,197 reads of 101 bp each were obtained for 224 individuals from AKA in the Red Sea. As a conservative measure, 29 individuals from the APS were discarded due to low read recovery (<500,000, likely due to DNA degradation resulting from poor preservation), which comprised only 0.4% of all reads. A total of 1,422,802 loci were built into the catalogue, with a coverage depth of 4×–55× and an average of 14.4×. After further filtering, a total of 19,544 loci were retained, which contained at least one randomly selected SNP present in over 95% of individuals in the sample with a minor allele frequency of at least 5% and a maximum heterozygosity of 60%. These gave genotypes for 193 individuals (25 from the APS and 168 from the RCS), with less than 5% missing alleles and a minimum coverage depth of 6x per locus. From these and for the analysis of population structure and sibship assignment, we selected the 300 most informative and adequate loci for sibship analyses, all in Hardy–Weinberg and linkage equilibrium, with a minimum minor allele frequency of 0.3.

### 3.2 Population genetic statistics and Bayesian clustering analysis

**FIGURE 3:**
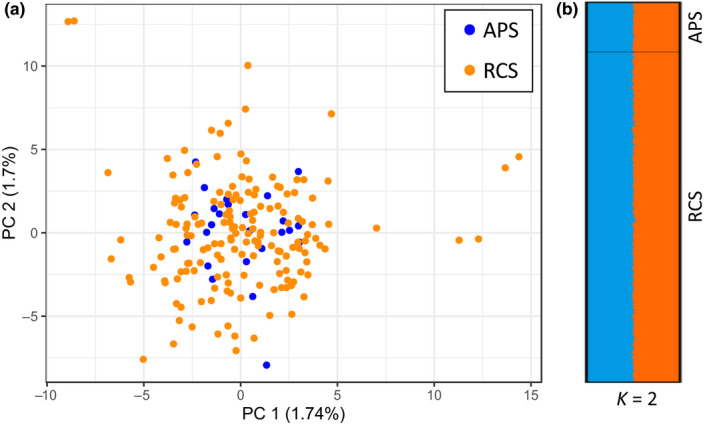
Results of the Principal Component Analysis (PCA, in R, panel a) and Bayesian clustering analyses (barplot, in STRUCTURE, panel b) using 300 independent SNP loci (ddRAD sequencing data) of *Dascyllus abudafur* from the north central Red Sea. The two samples compared are 25 adult fish (the adult population sample, APS) and a LED powered light trap sample comprising 168 recruiting larvae (the recruiting cohort sample, RCS), at one particular reef, Al‐Karrah. The PCA (in a) gives the results for the first (*x*‐axis) and second (*y*‐axis) principal components (PC), and their respective explanatory contribution in percent, while the STRUCTURE barplot (in b) gives the proportion of assignment of each individual to either one or the other of two putative genetic clusters (*K* = 2). Each horizontal line of the barplot represents one individual within the sample (either APS or RCS), and each color one of the two putative Ks

Using 300 independent SNP loci, the expected heterozygosity was high and similar for both analyzed samples, the RCS and APS (He = 0.48 and 0.47, respectively, Table 1), as expected from the criteria chosen for the loci selection to build the dataset. The PCA of genotypes of both samples (APS and RCS) did not display segregations between the two, and the first three axes were only able to explain 5.1% of the total variation of the data (PC 1:1.74%; PC 2:1.7%, and PC 3:1.65%; Figure 3a). Bayesian clustering analysis (STRUCTURE) was also not able to detect any population structure within the two samples (see *K* = 2, Figure 3b). These results were coherent with the lack of genetic differentiation between APS and RCS (*F*_ST_ = 0.002, *p* = .601; *F’*_ST_ = 0.003, *p* = .339, Table 1).

**TABLE 1:** Summary of principal population genetic statistics of ddRAD sequencing SNPs data for the two analyzed samples (APS: Adult population sample and RCS: Recruiting cohort sample) of *Dascyllus abudafur* from the north central Red Sea calculated in GenAlEx v.6.501. The number of individuals (Sample size) used for the analyses is provided in the first column. Effective number of alleles (*N*_e_), observed (*H*_obs_) and expected heterozygosity (*H*_exp_), and fixation indices (*F*_ST_, *F’*_ST_, and *F*_IS_) follow either overall or for both samples

	Sample size	*N*_e_	*H*_obs_	Hexp	*F*_ST_	*F’*_ST_	*F*_IS_
APS	25	1.894	0.455	0.470	—	—	0.032
RCS	168	1.909	0.466	0.480	—	—	0.028
					0.002	0.003	
*p*‐Value	—	—	—	—	.609	.339	.001

### 3.3 Sibship assignment

**FIGURE 4:**
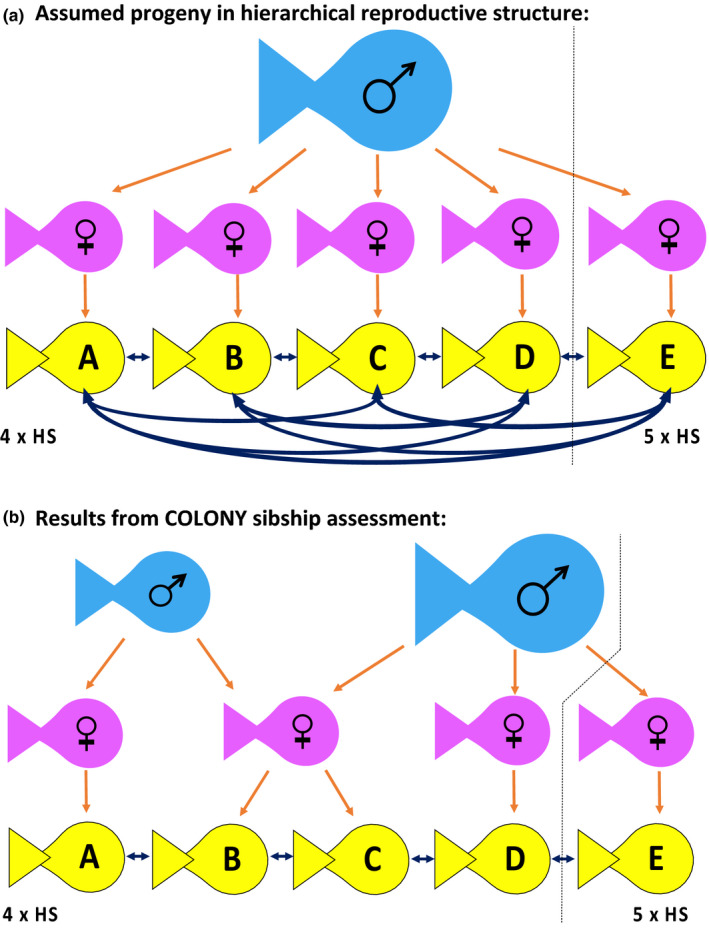
Theoretical (panel a) and empirical (panel b) sibship assignments among quadruplets (4 × HS) and quintuplets (5 × HS) of *Dascyllus abudafur* from the north central Red Sea. Panel a exemplifies the typical hierarchical reproductive structure found in the species, where one dominant single male (blue fish silhouette) produces progeny (yellow fish silhouettes) with multiple females (pink fish silhouettes) in a single colony. Panel b shows the empirical results we found using the program COLONY to infer sibship assignments, which essentially deviate from the expected hierarchical reproductive structure and rather suggest the presence of at least two reproducing males in a single colony. Orange arrows represent the pedigree of the progeny (yellow fish) and the black connecting arrows between the progeny represent the half sibling assignment. The dashed lines (in panels a and b) split the sibship results among either four (4×) or five (5×) half siblings (HS), which was the highest sibship assignment found among our data

Siblings within the RCS were identified using COLONY. From the 168 genotyped recruits, three pairs of full siblings had a probability of assignment equal to 1.0; while from the 126 pairs of half siblings, only 45 assignments had a probability above 0.75 and were accepted as true half siblings. Of these, 18 assignments had a probability of assignment of 0.95 or higher. From these calculations we concluded that the maximum possible number of source colonies providing the RCS sample is 120 (since among the 168 recruits at least 48 were coming from the same colony (i.e., full siblings + half siblings), thus indicating that a maximum of 120 (= 168 – 48, = C_max_) colonies produced our RCS, which is 29% fewer colonies than expected if all recruits would be traveling independently (i.e., each coming from an independent colony of *D. abudafur*). The full siblings and half siblings were comprised of 32 putative families as follows: 3 full siblings (one within a quadruplet assignment and the other two as single pairs), 18 half sibling pairs, 10 triplets, 1 quadruplet, and 1 quintuplet sibling‐group; which means that at least 79 recruits were arriving with a minimum of one kin/sibling to their new home reef (i.e., 47% of the RCS was recruiting with its kin). The remaining 89 recruits (53% of the total RCS) either arrived alone or their siblings were not captured with our light trap. Among the sibling assignments larger than triplets, deviations from the typical hierarchical reproductive colony structure of *D. abudafur* were identified. For instance, among the quadruplet and quintuplet related individuals, only three of them were half siblings at a time (Figure 4, panel B: “4 × HS” and “5 × HS,” respectively). This means that more than one male must have fertilized the eggs of that colony (see Figure 4, panel A vs. panel B).

### 3.4 Statistical significance of kinship results and data mining

**FIGURE 5:**
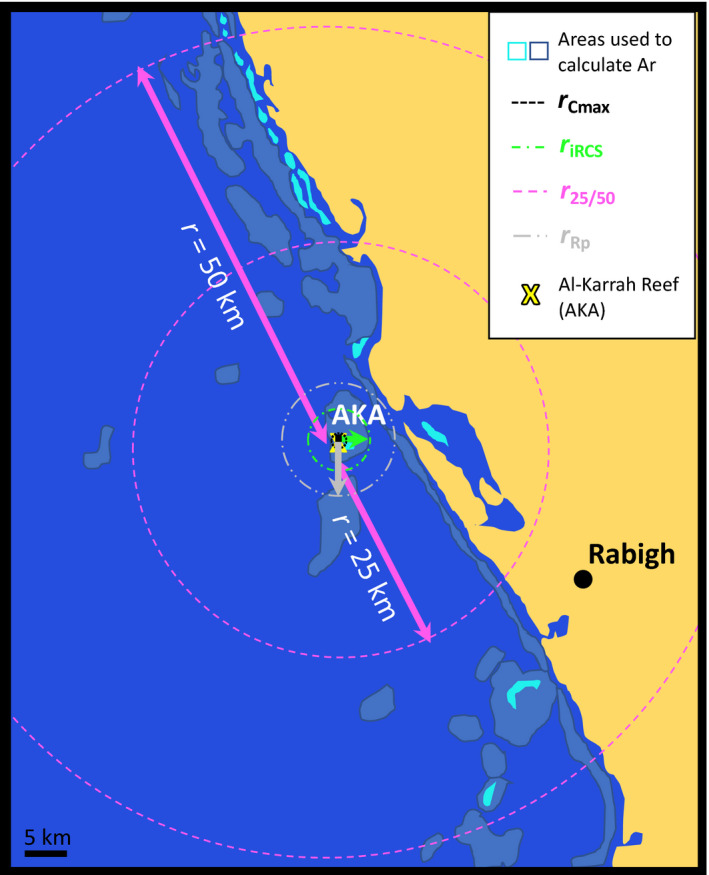
Map of the coastal area around our sampling site, Al Karrah reef (AKA, north central Saudi Arabian Red Sea), near the city of Rabigh, displaying the area of putative radii providing larvae of *Dascyllus abudafur* that may recruit to AKA. The area of each radius (A_r_) used to estimate the number of colonies providing aforementioned recruits, solely included potential habitat which is based on bathymetry and therefore only includes the coastal sections in the two lighter blue colors given in the figure's legend. The legend further indicates the radii corresponding to the different colored arrows and circles around AKA (marked with a yellow cross). Their exact sizes are, from smallest to largest: 1.066 km (r_Cmax_, in black), 1.09 km (r_iRCS_, in green), 4.31 km (r_Rp_, in gray), and 25 km and 50 km (r_25/50_, in pink). Details to their calculations can be found in Table 2

Monte Carlo simulations were run for various TRC_r_s. The different radii associated to each of the following TRCs are drawn in Figure 5 for scale. Random draws of 168 individuals (cRCS) out of a TRC containing 6 siblings (= avg. R_Cd_) per recruiting colony (= C_A_ × F_R%_), from reefs from a r25 km radius around AKA resulted in an average of 3.49 sibling pairs (SP_r25_), 0.04 sibling triplets, and 0 sibling quadruplets; and about half for r50: i.e., 1.47 sibling pairs (SP_r50_), 0.01 triplets, and 0 quadruplets. With these results, the minimum radius (r_Rp_) that could give us the results obtained with COLONY was estimated to be 4.31 km, equivalent to an area of 58.37 km^2^ (i.e., A_Rp_ = r_Rp_^2^ × π); and a TRC_rRp_ = R_Cd_ × C_A_ × F_R%_ × A_RCP_ = 5,911 total recruits. This was calculated following two assumptions: (e) ½ × r50 = r25; (f) SP_r25_ = SP_r50_ × Rp; for which, in theory, the sibling pair assignment increased over twofold, every time the radius was halved by Rp = 3.49/1.47 = 2.37. COLONY found 48 sibling pairs (SP_RCP_) within our RCS. So (g) n × Rp × SP_r25_ = SP_RCP_ gives the number of how many times (n), the radius has to be halved to draw 48 sibling pairs, randomly. Therefore, *n* = SP_RCP_ × Rp^‐1^ × SP_r25_^‐1^ = 48/8.26 = 5.81; the theoretical radius (r_Rp_) 1/*n* × r25 = 25 km/5.8 = 4.31 km.

The COLONY results also indicate that a maximum of 120 colonies (= C_max_) provide our RCS. Assuming that each one of these colonies produces an average R_Cd_ of 6, and if every single colony that built our TRC was represented within our light trap sample (RCS), then the TRC (coming from only 120 colonies) would be of R_Cd_ × C_max_ = 6 × 120 = 720 (= TRC_Cmax_), which is much lower than the TRC_rRp_ (from above) and equivalent to an area of A_Cmax_ = TRC_Cmax_/(R_Cd_ × C_A_ × F_R%_) = 720/ (6 × (102/km^2^) × 0.33) = 3.57 km^2^ (for: TRC_Cmax_ = R_Cd_ × C_A_ × F_R%_ × A_Cmax_) and a radius of r_Cmax_ = √(A_Cmax_/*π*) = 1.066 km.

The simulations to pick random samples of 168 and assess the sibling pairs were also run for TRC_rRp_ and TRC_Cmax_, for which the former gave a SP_Rp_ of 11, 0.41 triplets, and 0.01 quadruplets; and for TRC_Cmax_: 34 SP_Cmax_, 13.71 triplets, and 3.08 quadruplets, 0.37 quintets, and 0.02 sextets. With the second programmed tool, our data was given a score value of 142 and after 100,000 random picks that score was only observed in 0.015% of the random picks within TRC_rRp_. This latter result indicates how rare our empirical sibship results were even within a relatively small TRC. Furthermore, the tool computed that the TRC_riRCP_ would have to be as little as 750 recruits only (i.e., coming from a maximum of 125 colonies and a radius of 1.09 km (r_iRCP_), Table 2) to have a 95% chance to find sibship assignments similar to those given by our data when picking 168 individuals randomly. Altogether, our results highlight the significance of our data and its potential to reject the null hypothesis that the recruiting sample travels as a randomly mixed cohort of larvae and not as groups of close kin.

**TABLE 2:** Calculated values from literature and discussions with experts regarding key biological features of *Dascyllus abudafur* for computational inferences. The most important abbreviations mentioned in the Results section are listed together with their respective meaning (and calculations as explained in the Section 2)

Abbreviation	Meaning and source	Value
AKA	Al‐Karrah Reef: sampling site in the central Red Sea, off Saudi Arabia.	N22°56.265, E38°45.943
RCS	Recruiting Cohort Sample: 171 individuals of recruiting larvae of *Dascyllus abudafur* sampled with an LED powered Light Trap at AKA. SNPs of 168 of these were used for genetic analyses and all 171 for pelagic larval duration (PLD) assessment.	168
APS	Adult Population Sample: 53 juvenile and adult specimens collected at 15m depth from 4 colonies at AKA. SNPs of 38 were used for genetic analyses and 11 for PLD assessment.	38
SP	Sibling Pair assignment: either as calculated by COLONY within our RCS or given for random picks out of a computed RCS (cRCS) by an application written in PYTHON.	Values are (c)RCS specific
TRC	Theoretical Total Recruiting Cohort: inferred either from literature or from computational iterations.	Values vary, see below
cRCS	Computed Recruiting Cohort Sample: randomly picked RCS of the size *N* = 168 (equal to the empirical RCS) from inferred TRCs.	Values vary, see below
TRC_r25/50_	TRC composed of recruits originating from larvae produced within a radius of 25 or 50 km of suitable habitat around AKA. Its value is calculated with TRC_r_ = R_Cd_ × C_A_ × FR_%_ × A_r_. TRC_r25_: has an average of 3.49 SP_r25_, 0.04 sibling triplets, and 0 sibling quadruplets; which is about half for TRC_r50_: 1.47 SP_r50_, 0.01 triplets, and 0 quadruplets.	19,445 (r25) 47,033 (r50)
R_Cd_	The daily number of larvae produced by an average colony of *D. abudafur* that will survive the pelagic larval duration (PLD) and putatively, successfully recruit to a new home reef.	6
C_A_	The number of *D. abudafur* colonies per area of suitable habitat in the Red Sea (from averaged 50 m × 2 m transect data, from reefs in the central Red Sea). C_A_ = Da/At.	102/km^2^
F_R%_	The percentage of simultaneously reproductive active females in an average population of *D. abudafur* (taken from Mizushima et al., 2000).	0.33
A_r_	The area of suitable habitat available for *D. abudafur* in a chosen radius with putative parental source colonies. A_r_ = L × W.	192 km^2^ (r25) 464.4 km^2^ (r50)
E	The average number of eggs per colony of *D. abudafur* (following Wong, Fauvelot, Planes, and Buston 2012).	803
H	The hatch rate per clutch of *D. abudafur* (anything between 1 and 0 is possible as agreed among experts: Giacomo Bernardi, Ricardo Beldade, and Suzanne Mills).	0.5
Hd_%_	The percentage of eggs of *D. abudafur* that hatched per day (Mizushima et al., 2000).	0.5
S	Survival rate of *D. abudafur* larvae after the PLD (in agreement with Giacomo Bernardi and Ricardo Beldade)	From 0.01 to 0.05
At	Unit of belt transects (50 m × 2 m) used to assess colony density of *D. abudafur*.	0.1 km^2^
Da	Average number of colonies of *D. abudafur* in 0.1 km^2^ habitat in the Red Sea.	10.23
L	The trajectory of potential habitat for *D. abudafur* around AKA for the two chosen radii (r25 and r50), measured with Google Earth satellite imagery.	160 km (r25) 387 km (r50)
W	Average width of *D. abudafur* habitat following bathymetry measurements from central Red Sea reefs (provided by Maha Khalil) and using a depth range from 1 m to 19 m as suitable habitat.	1.2 km
Rp	The rate at which the sibling pair assignments in a cRCS (from a TRC_r_ of *D. abudafur*) increases as the radius is halved. Calculated from SP_r25_ = SP_r50_ × Rp.	2.37
Cmax	Maximum number of colonies that provided the recruits of *D. abudafur* captured in the light trap at AKA.	120
*n*	The number of times a radius of 25 km (r25) has to be halved in order to draw as many sibling pairs (SPs) as the ones calculated by COLONY for our empirical RCS of *D. abudafur*. *n* = SP_RCS_ × Rp^−1^ × SP_r25_^−1^	5.811
r_Rp_	The maximum source radius for a putative *D. abudafur* TRC to achieve similar results in a cRCS to those given by COLONY for our empirical RCS (estimated from the Rp calculated from the resulting sibling pair assignments for TRC_r25_ and TRC_r50_).	4.31 km
r_Cmax_	The theoretical radius around AKA in which one could find a maximum of 120 colonies of *D. abudafur* that could produce larvae to recruit to AKA. r_Cmax_ = √(A_Cmax_/ π).	1.07 km
A_Cmax_	The Cmax area of *D. abudafur* habitat with colonies recruiting to AKA. A_Cmax_ = TRC_Cmax_/(R_Cd_ × C_A_ × F_R%_).	3.57 km^2^
TRC_rRp_	The TRC from the maximum possible radius (rRp = 4.31 km, inferred from the results of TRCr25 and TRCr50) to achieve by random, sibship results similar to those from the empirical RCS. SP_Rp_ of 11, 0.41 triplets, and 0.01 quadruplets.	5,911
TRC_Cmax_	The TRC that results for the maximum number of colonies recruiting to AKA according to the RCS sibship analysis by COLONY. 34 SP_Cmax_, 13.71 triplets, and 3.08 quadruplets, 0.37 quintets, and 0.02 sextets.	720
TRC_riRCS_	The TRC of the maximum number of colonies (C_riRCS_ = 125; riRCS = 1.09 km) of *D. abudafur* that can be producing larvae recruiting to AKA in order to achieve with 95% chance a similar SP_RCS_ (=48) to that found among the recruits of our empirical RCS.	750

## 4 DISCUSSION

In this study, we have clear evidence of a significantly high presence of siblings within the cohort of a single recruitment event. Despite the fact that we cannot follow these larvae throughout their PLD, our results strongly suggest that larvae from *Dascyllus abudafur* stay with their kin after hatching, traveling together throughout their pelagic life stage until they reach their settlement habitat. Within the sample of 168 larval recruits captured in a single light trap during one single night at Al‐Karrah Reef, ddRAD analyses indicated that at least 47% of these new recruits had arrived with a minimum of one relative and up to a group of five half siblings was detected within this spatially and temporally narrow sample. Other studies on kinship assessment have previously reported the presence of siblings among coral reef fishes within a recruiting cohort. The percentage of sibling assignments of aforementioned studies were much lower (6%–12%) but were based on the sampling of recently settled recruits or juveniles (Bernardi et al., 2012; Herrera et al., 2016) and not on new recruiting larvae. Further, the statistical significance of their findings was not addressed. Using permutation‐based inferences, our study addresses the significance of recruitment with siblings after the PLD and potential cohesive dispersal. With our approach, we found our sibship results to be nonrandom, implying that the fishes were actively traveling together in the pelagic environment with their kin. For the light trap sample to be a random sample of possible larval output, we infer that the source must only be an improbably very small area (approx. 1 to 4 km of radius). Given what we know about connectivity in the Red Sea (Nanninga, 2013; Nanninga et al., 2015; Raitsos et al., 2017), such a tiny source area seems unlikely (see Fig. 6 for scale). Hence, even if most assumptions implemented in our computations are unrealistic, we provide an example of how to approach robust predictions when still lacking much data in a nonmodel organism. Based on the evidence, we assume our observations to be the result of an innate behavioral trait of schooling among kin (in *D. abudafur*) rather than a simple stochastic occurrence, although hydrodynamic processes such as packet entrainment may also be acting upon the dispersal of larvae. We further have evidence of potential “sneaker” males and/or the presence of more than one reproducing male per colony, as the genetic kinship among the recruiting quadruplets and quintuplets indicated that these were the progeny of at least two males (as opposed to the expected haremic half siblings from different mothers but the same father, see Figure 4). In terms of genetic composition, the new recruits of our study were providing nearly the same genetic diversity contained in the adult population sample. Both samples (that of the recruiting cohort and the adult population samples) had high heterozygosity, which suggests a large, well‐mixed, and well‐established *D. abudafur* population in the north central Red Sea, rather than the presence of strong selective sweeps and restricted geneflow. Hence, even though larvae may travel together with their siblings, the ultimate genetic exchange between sources of recruiting fishes seems to be large and stable.

### 4.1 Why are closely related larvae traveling together?

Little is known about schooling in coral reef fish larvae. Based on studies of migratory fishes from temperate regions, one could infer that schooling may have evolved to aid navigation through collective movement, especially when it comes to finding a way back from the pelagic to the restricted reef habitat. In particular among coral reef fish larvae, schooling emerges as a vital innate behavior to succeed when relying on finding targets as small as coral reefs. Schooling among your kin could further increase the ability to find suitable food as well as chemical and physical cues for navigation (for example, the ‘many wrongs principle’; Codling, Pitchford, & Simpson, 2007; Larkin & Walton, 1969; Simons, 2004). It could also decrease risk of predation (Handegard et al., 2012).

Moreover, traveling in schools comprised of their closest kin may have additional advantages for larvae. Collectively, they have higher chances of recognizing dangerous predators, identifying food sources and cues (Handegard et al., 2012), and navigating to a suitable habitat (Berdahl, Westley, Levin, Couzin, & Quinn, 2016; Couzin, 2007; Torney, Berdahl, & Couzin, 2011; Torney, Neufeld, & Couzin, 2009). In schools of fish, it has also been shown that the ability to sense odor cues increases with group size (Hall, Burton, Margrey, & Graves, 1982) and coral reef fish larvae particularly rely on such cues when recruiting home (Paris et al., 2013). Hence, schooling with your siblings may not only be advantageous but also the easiest alternative, because these are the nearest kin to be found right after hatching. The likelihood of sibling larvae to school could therefore also differ between reproductive strategies (broadcast spawners vs. benthic brooders) and among environmental conditions (such as current strength and food availability). Schooling has also been found to have hydrodynamic benefits (Herskin & Steffensen, 1998; Ross, Backman, & Limburg, 1992) for which it might be worth waiting for siblings to hatch not only for it to be easier to swim in a certain direction but also to save energy when resources are scarce. This may not be possible if currents are too strong, or if the larvae hatch in the pelagic as a result of broadcast spawning, which warrants further research.

### 4.2 What are the consequences of schooling with kin during the pelagic larval duration (PLD) for population dynamics?

Assuming that only a small proportion of the available gene pool is transported to the population by one recruitment event (“sweepstakes effect”; Hedgecock, 1994), a single cohort is expected to have less genetic diversity than that found among cohorts (Hedgecock, Barber, & Edmands, 2007). Indeed, significant changes in allele frequencies of larvae and recruits have been documented from one sampling time to the next (Moberg & Burton, 2000; Selkoe, Gaines, Case lle, & Warner, 2006; Toonen & Pawlik, 2001). Schooling with your kin and recruiting with your siblings may further increase the “sweepstakes effect.” This could eventually lead to population differentiation if the source of recruits is restricted to a single location with low genetic diversity over a couple of generations. However, in population genetics, the exchange and successful reproduction of a single individual from different reefs per generation is already enough to keep connectivity between populations and hinder genetic drift (Hellberg, Burton, Neigel, & Palumbi, 2002; Thorrold et al., 2002). Even though we find a scenario in which siblings of *D. abudafur* are actively schooling together, a single recruitment event was able to provide as much of the genetic richness present in the adult population sample. This can be interpreted as a highly panmictic population, in which a large number of colonies are reproducing together and providing high connectivity between reefs, despite cohesive dispersal. We further propose that while it is a good strategy to codisperse, the larvae are not settling together with their kin into a single colony, countering interbreeding. But the exact implications our results may have on the genetic structure of the *D. abudafur* population in the Red Sea still remains to be investigated. Sensitivity analyses combining theoretical and empirical values related to larval dispersal (e.g., dispersal kernels, in situ hydrodynamic measurements for particle movement modeling, mortality rates, and reproductive cycles) and including data from large‐ and small‐scale genetic studies (such as those in Saenz‐Agudelo et al., 2015; Schunter et al., 2019), would improve our approach and give further insight into the population dynamics of the species. Moreover, it would be interesting to investigate whether cohesive dispersal with siblings is also a distinguishing behavioral larval trait among other coral reef fishes and maybe another crucial factor determining dispersal, population structure, and biogeographic ranges. While it is hard to define whether our finding of cohesive dispersal of siblings among coral reef fishes is an exceptional or a common behavioral larval trait, we advocate for future studies to use similar suitable genetic markers to identify relationships among conspecific corecruiting larvae (sampled temporally punctual and in large numbers) and test this behavior among other coral reef fish species. The genetic relatedness among new recruits of coral reef fishes within a settling cohort will provide essential knowledge of mechanistic features of larval dispersal (Buston et al., 2009; Planes, 2002; Selkoe et al., 2006), crucial for the management of marine populations.

## CONFLICT OF INTEREST

The authors declare no competing interests.

## AUTHOR CONTRIBUTIONS

**Vanessa Robitzch:** Conceptualization (lead); Data curation (lead); Formal analysis (lead); Investigation (lead); Methodology (lead); Project administration (equal); Resources (lead); Software (lead); Supervision (lead); Validation (lead); Visualization (lead); Writing‐original draft (lead); Writing‐review & editing (lead). **Pablo Saenz‐Agudelo:** Data curation (equal); Formal analysis (equal); Investigation (equal); Methodology (equal); Software (equal); Supervision (equal); Writing‐original draft (equal); Writing‐review & editing (equal). **Michael L. Berumen:** Conceptualization (equal); Funding acquisition (lead); Project administration (equal); Resources (equal); Supervision (equal); Writing‐original draft (equal); Writing‐review & editing (equal).

## Supporting information

Supplementary Material

## Data Availability

‐ Script for Monte Carlo simulations: https://github.com/HexTree/fish‐siblings/blob/master/fish_sim.py
‐ Sampling coordinates as given in the text (Figure 1 and Table 2) ‐ Genotypes for all individuals included in this study (based on Single Nucleotide Polymorphism, SNP data): Dryad https://doi.org/10.5061/dryad.h44j0zpgx.

## References

[ece36533-bib-0001] Almany, G. R. , Hamilton, R. J. , Bode, M. , Matawai, M. , Potuku, T. , Saenz‐Agudelo, P. , … Jones, G. P. (2013). Dispersal of grouper larvae drives local resource sharing in a coral reef fishery. Current Biology, 23, 626–630. 10.1016/j.cub.2013.03.006 23541728

[ece36533-bib-0002] Almany, G. R. , Planes, S. , Thorrold, S. R. , Berumen, M. L. , Bode, M. , Saenz‐Agudelo, P. , … Jones, G. P. (2017). Larval fish dispersal in a coral‐reef seascape. Nature Ecology and Evolution, 1, 1–7. 10.1038/s41559-017-0148 28812625

[ece36533-bib-0003] Avise, J. C. , & Shapiro, D. Y. (1986). Evaluating kinship of newly settled juveniles within social groups of the coral reef fish *Anthias squamipinnis* . Evolution, 40, 1051–1059. 28556214 10.1111/j.1558-5646.1986.tb00572.x

[ece36533-bib-0004] Berdahl, A. , Westley, P. A. H. , Levin, S. A. , Couzin, I. D. , & Quinn, T. P. (2016). A collective navigation hypothesis for homeward migration in anadromous salmonids. Fish and Fisheries, 17, 525–542. 10.1111/faf.12084

[ece36533-bib-0005] Bernardi, G. , Beldade, R. , Holbrook, S. J. , & Schmitt, R. J. (2012). Full‐sibs in cohorts of newly settled coral reef fishes. PLoS One, 7, e44953 10.1371/journal.pone.0044953 23028700 PMC3441696

[ece36533-bib-0006] Berumen, M. L. , Almany, G. R. , Planes, S. , Jones, G. P. , Saenz‐Agudelo, P. , & Thorrold, S. R. (2012). Persistence of self‐recruitment and patterns of larval connectivity in a marine protected area network. Ecology and Evolution, 2, 444–452. 10.1002/ece3.208 22423335 PMC3298954

[ece36533-bib-0007] Borsa, P. , Sembiring, A. , Fauvelot, C. , & Chen, W.‐J. (2014). Resurrection of Indian Ocean humbug damselfish, *Dascyllus abudafur* (Forsskål) from synonymy with its Pacific Ocean sibling, *Dascyllus aruanus* (L.). Comptes Rendus Biologies, 337, 709–716. 10.1016/j.crvi.2014.09.001 25433563

[ece36533-bib-0008] Buston, P. M. , Fauvelot, C. , Wong, M. Y. L. , & Planes, S. (2009). Genetic relatedness in groups of the humbug damselfish *Dascyllus aruanus*: Small, similar‐sized individuals may be close kin. Molecular Ecology, 18, 4707–4715. 19845858 10.1111/j.1365-294X.2009.04383.x

[ece36533-bib-0009] Catchen, J. M. , Amores, A. , Hohenlohe, P. , Cresko, W. , & Postlethwait, J. H. (2011). Stacks: Building and genotyping loci *de novo* from short‐read sequences. Genes, Genomes, Genetics, 1, 171–182. 22384329 10.1534/g3.111.000240 PMC3276136

[ece36533-bib-0010] Coates, D. (1982). Some observations on the sexuality of humbug damselfish, *Dascyllus aruanus* (Pisces, Pomacentridae) in the field. Zeitschrift für Tierpsychologie, 59, 7–18. 10.1111/j.1439-0310.1982.tb00328.x

[ece36533-bib-0011] Codling, E. A. , Pitchford, J. W. , & Simpson, S. D. (2007). Group navigation and the “many‐wrongs principle” in models of animal movement. Ecology, 88, 1864–1870. 10.1890/06-0854.1 17645033

[ece36533-bib-0012] Couzin, I. (2007). Collective minds. Nature, 445, 715 10.1038/445715a 17301775

[ece36533-bib-0013] Eble, J. V. A. , Toonen, R. J. , & Bowen, B. W. (2009). Endemism and dispersal: Comparative phylogeography of three surgeonfishes across the Hawaiian Archipelago. Marine Biology, 156, 689–698. 10.1007/s00227-008-1119-4

[ece36533-bib-0014] Fricke, H. W. , & Holzberg, S. (1974). Social units and hermaphroditism in a pomacentrid fish. Naturwissenschaftern, 61, 367–368. 10.1007/BF00600312

[ece36533-bib-0015] Gerlach, G. , Atema, J. , Kingsford, M. J. , Black, K. P. , & Miller‐Sims, V. (2007). Smelling home can prevent dispersal of reef fish larvae. Proceedings of the National Academy of Sciences of the United States of America, 104, 858–863. 10.1073/pnas.0606777104 17213323 PMC1783404

[ece36533-bib-0016] Gillespie, A. (2009). Group dynamics and interactions between two cohabiting damselfishes, *Dascyllus aruanus* and *Pomacentrus moluccensis* . Stanford Undergraduate Research Journal, 82–86. https://web.stanford.edu/group/journal/cgi‐bin/wordpress/wp‐content/uploads/2012/09/Gillespie_NatSci_2009.pdf

[ece36533-bib-0017] Hall, L. W. Jr , Burton, D. T. , Margrey, S. L. , & Graves, W. C. (1982). A comparison of the avoidance responses of individual and schooling juvenile Atlantic menhaden, *Brevoortia tyrannus* subjected to simultaneous chlorine and delta T conditions. Journal of Toxicology and Environmental Health, 10, 1017–1026. 7161833 10.1080/15287398209530313

[ece36533-bib-0018] Handegard, N. O. , Boswell, K. M. , Ioannou, C. C. , Leblanc, S. P. , Tjøstheim, D. B. , & Couzin, I. D. (2012). Report the dynamics of coordinated group hunting and collective information transfer among schooling prey. Current Biology, 22, 1213–1217. 10.1016/j.cub.2012.04.050 22683262

[ece36533-bib-0019] Hedgecock, D. (1994). Does variance in reproductive success limit effective population sizes of marine organisms? In Beaumont M. A. (Ed.), Genetics and evolution of aquatic organisms (pp. 122–134). London, UK: Chapman & Hall.

[ece36533-bib-0020] Hedgecock, D. , Barber, P. H. , & Edmands, S. (2007). Genetic approaches to measuring connectivity. Oceanography, 20, 70–79. 10.5670/oceanog.2007.30

[ece36533-bib-0021] Hellberg, M. E. , Burton, R. S. , Neigel, J. E. , & Palumbi, S. R. (2002). Genetic assessment of connectivity among marine populations. Bulletin of Marine Science, 70, 273–290.

[ece36533-bib-0022] Herrera, M. , Nanninga, G. B. , Planes, S. , Jones, G. P. , Thorrold, S. R. , Saenz‐Agudelo, P. , … Berumen, M. L. (2016). Seascape and life‐history traits do not predict self‐recruitment in a coral reef fish. Biology Letters, 12, 4–7. 10.1098/rsbl.2016.0309 PMC5014023 27512132

[ece36533-bib-0023] Herskin, J. , & Steffensen, J. F. (1998). Energy savings in sea bass swimming in a school: Measurements of tail beat frequency and oxygen consumption at different swimming speeds. Journal of Fish Biology, 53, 366–376. 10.1111/j.1095-8649.1998.tb00986.x

[ece36533-bib-0024] Jones, G. P. , Planes, S. , & Thorrold, S. R. (2005). Coral reef fish larvae settle close to home. Current Biology, 15, 1314–1318. 10.1016/j.cub.2005.06.061 16051176

[ece36533-bib-0025] Kingsford, M. J. , Leis, J. M. , Shanks, A. , Lindeman, K. C. , Morgan, S. G. , & Pineda, J. (2002). Sensory environments, larval abilities and local self‐recruitment. Bulletin of Marine Science, 70, 309–340.

[ece36533-bib-0026] Kopelman, N. M. , Mayzel, J. , Jakobsson, M. , Rosenberg, N. A. , & Mayrose, I. (2015). Clumpak: A program for identifying clustering modes and packaging population structure inferences across K. Molecular Ecology Resources, 15, 1179–1191. 25684545 10.1111/1755-0998.12387 PMC4534335

[ece36533-bib-0027] Larkin, P. A. , & Walton, A. (1969). Fish school size and migration. Journal of the Fisheries Board of Canada, 26, 1372–1374. 10.1139/f69-121

[ece36533-bib-0028] Lecchini, D. , & Nakamura, Y. (2013). Use of chemical cues by coral reef animal larvae for habitat selection. Aquatic Biology, 19, 231–238. 10.3354/ab00532

[ece36533-bib-0029] Leis, J. (1991). The pelagic stage of reef fishes: The larval biology of coral reef fishes In Sale P. F.(Ed.), The ecology of fishes on coral reefs (pp. 183–230). San Diego, CA: Academic Press.

[ece36533-bib-0030] Leis, J. M. (2006). Are larvae of demersal fishes plankton or nekton? Advances in Marine Biology, 51, 59–141. 10.1016/S0065-2881(06)51002-8 16905426

[ece36533-bib-0031] Leis, J. M. , & McCormick, M. I. (2002). The biology, behavior, and ecology of the pelagic, larval stage of coral reef fishes In Sale P. F. (Ed.), The ecology of fishes on coral reefs (pp. 171–200). San Diego, CA: Academic Press Inc.

[ece36533-bib-0032] Lischer, H. E. L. , & Excoffier, L. (2012). PGDSpider: An automated data conversion tool for connecting population genetics and genomics programs. Bioinformatics (Oxford, England), 28, 298–299. 10.1093/bioinformatics/btr642 22110245

[ece36533-bib-0033] Madduppa, H. H. , Timm, J. , & Kochzius, M. (2014). Interspecific, spatial and temporal variability of self‐recruitment in anemonefishes. PLoS One, 9, e90648 10.1371/journal.pone.0090648 24587406 PMC3938785

[ece36533-bib-0034] Mastretta‐Yanes, A. , Arrigo, N. , Alvarez, N. , Jorgensen, T. H. , Piñero, D. , & Emerson, B. C. (2014). Data from: RAD sequencing, genotyping error estimation and de novo assembly optimization for population genetic inference. Dryad Digital Repository, 10.5061/dryad.g52m3 24916682

[ece36533-bib-0035] Mizushima, N. , Nakashima, Y. , & Kuwamura, T. (2000). Semilunar spawning cycle of the humbug damselfish *Dascyllus aruanus* . Journal of Ethology, 18, 105–108. 10.1007/s101640070008

[ece36533-bib-0036] Moberg, P. E. , & Burton, R. S. (2000). Genetic heterogeneity among adult and recruit Red Sea urchins, *Strongylocentrotus franciscanus* . Marine Biology, 136, 773–784. 10.1007/s002270000281

[ece36533-bib-0037] Montgomery, J. C. , Tolimieri, N. , & Haine, O. S. (2001). Active habitat selection by pre‐settlement reef fishes. Fish and Fisheries, 2, 261–277. 10.1046/j.1467-2960.2001.00053.x

[ece36533-bib-0038] Mouritsen, H. , Atema, J. , Kingsford, M. J. , & Gerlach, G. (2013). Sun compass orientation helps coral reef fish larvae return to their natal reef. PLoS One, 8, e66039 10.1371/journal.pone.0066039 23840396 PMC3694079

[ece36533-bib-0039] Nanninga, G. B. (2013). Merging approaches to explore connectivity in the anemonefish, Amphiprion bicinctus, along the Saudi Arabian coast of the Red Sea. PhD thesis, King Abdullah University of Science and Technology.

[ece36533-bib-0040] Nanninga, G. B. , Saenz‐Agudelo, P. , Zhan, P. , Hoteit, I. , & Berumen, M. L. (2015). Not finding Nemo: Limited reef‐scale retention in a coral reef fish. Coral Reefs, 34, 383–392. 10.1007/s00338-015-1266-2

[ece36533-bib-0041] Paris, C. B. , Atema, J. , Irisson, J.‐O. , Kingsford, M. , Gerlach, G. , & Guigand, C. M. (2013). Reef odor: A wake up call for navigation in reef fish larvae. PLoS One, 8, e72808 10.1371/journal.pone.0072808 24015278 PMC3755995

[ece36533-bib-0042] Paris, C. B. , & Cowen, R. K. (2004). Direct evidence of a biophysical retention mechanism for coral reef fish larvae. Limnology and Oceanography, 49, 1964–1979. 10.4319/lo.2004.49.6.1964

[ece36533-bib-0043] Peakall, R. , & Smouse, P. E. (2006). GenAlEx 6: Genetic analysis in Excel. Population genetic software for teaching and research. Molecular Ecology Notes, 6, 288–295. 10.1111/j.1471-8286.2005.01155.x PMC3463245 22820204

[ece36533-bib-0044] Peakall, R. , & Smouse, P. E. (2012). GenAlEx 6.5: Genetic analysis in Excel. Population genetic software for teaching and research ‐ an update. Bioinformatics (Oxford, England), 28, 2537–2539. 10.1093/bioinformatics/bts460 PMC3463245 22820204

[ece36533-bib-0045] Peterson, B. K. , Weber, J. N. , Kay, E. H. , Fisher, H. S. , & Hoekstra, H. E. (2012). Double digest RADseq: An inexpensive method for de novo SNP discovery and genotyping in model and non‐model species. PLoS One, 7, e37135 10.1371/journal.pone.0037135 22675423 PMC3365034

[ece36533-bib-0046] Pinsky, M. L. , Palumbi, S. R. , Andréfouët, S. , & Purkis, S. J. (2012). Open and closed seascapes: Where does habitat patchiness create populations with high fractions of self‐recruitment? Ecological Applications, 22, 1257–1267. 10.1890/11-1240.1 22827133

[ece36533-bib-0047] Planes, S. (2002). Biogeography and larval dispersal inferred from population genetic analysis In Sale P. F. (Ed.), Coral reef fishes (pp. 201–220). San Diego, CA: Academic Press Inc.

[ece36533-bib-0048] Pritchard, J. K. , Stephens, M. , & Donnelly, P. (2000). Inference of population structure using multilocus genotype data. Genetics, 155, 945–959. 10835412 10.1093/genetics/155.2.945 PMC1461096

[ece36533-bib-0049] Raitsos, D. E. , Brewin, R. J. W. , Zhan, P. , Dreano, D. , Pradhan, Y. , Nanninga, G. B. , & Hoteit, I. (2017). Sensing coral reef connectivity pathways from space. Scientific Reports, 7, 9338 10.1038/s41598-017-08729-w 28839286 PMC5571014

[ece36533-bib-0050] Raymond, M. , & Rousset, F. (1995). GENEPOP (version 1.2): Population genetics software for exact tests and ecumenicism. Journal of Heredity, 86, 248–249. 10.1093/oxfordjournals.jhered.a111573

[ece36533-bib-0051] Robitzch, V. , Lozano‐Cortés, D. , Kandler, N. M. , Salas, E. , & Berumen, M. L. (2016). Productivity and sea surface temperature are correlated with the pelagic larval duration of damselfishes in the Red Sea. Marine Pollution Bulletin, 105(2), 566–574. 26654297 10.1016/j.marpolbul.2015.11.045

[ece36533-bib-0052] Ross, R. M. , Backman, T. W. H. , & Limburg, K. E. (1992). Notes: Group‐size‐mediated metabolic rate reduction in American shad. Transactions of the American Fisheries Society, 121, 385–390.

[ece36533-bib-0053] Roughgarden, J. , Iwasa, Y. O. H. , & Baxter, C. (1985). Demographic theory for an open marine population with space‐limited recruitment. Ecology, 66, 54–67. 10.2307/1941306

[ece36533-bib-0054] Rousset, F. (2008). GENEPOP’007: A complete re‐implementation of the GENEPOP software for Windows and Linux. Molecular Ecology Resources, 8, 103–106. 10.1111/j.1471-8286.2007.01931.x 21585727

[ece36533-bib-0055] Saenz‐Agudelo, P. , DiBattista, J. D. , Piatek, M. J. , Gaither, M. R. , Harrison, H. B. , Nanninga, G. B. , & Berumen, M. L. (2015). Seascape genetics along environmental gradients in the Arabian Peninsula: Insights from ddRAD sequencing of anemonefishes. Molecular Ecology, 24, 6241–6255. 10.1111/mec.13471 26577830

[ece36533-bib-0056] Schunter, C. , Garza, J. C. , Macpherson, E. , & Pascual, M. (2013). SNP development from RNA‐seq data in a nonmodel fish: How many individuals are needed for accurate allele frequency prediction? Molecular Ecology Resources, 14, 157–165. 10.1111/1755-0998.12155 23992151

[ece36533-bib-0057] Schunter, C. , Pascual, M. , Raventos, N. , Garriga, J. , Garza, J. C. , Bartumeus, F. , & Macpherson, E. (2019). A novel integrative approach elucidates fine‐scale dispersal patchiness in marine populations. Scientific Reports, 9, 10796 10.1038/s41598-019-47200-w 31346216 PMC6658486

[ece36533-bib-0058] Selkoe, K. A. , Gaines, S. D. , Caselle, J. E. , & Warner, R. R. (2006). Current shifts and kin aggregation explain genetic patchiness in fish recruits. Ecology, 87, 3082–3094. 17249233 10.1890/0012-9658(2006)87[3082:csakae]2.0.co;2

[ece36533-bib-0059] Selkoe, K. , & Toonen, R. (2011). Marine connectivity: A new look at pelagic larval duration and genetic metrics of dispersal. Marine Ecology Progress Series, 436, 291–305. 10.3354/meps09238

[ece36533-bib-0060] Selkoe, K. A. , Watson, J. R. , White, C. , Horin, T. B. , Iacchei, M. , Mitarai, S. , … Toonen, R. J. (2010). Taking the chaos out of genetic patchiness: Seascape genetics reveals ecological and oceanographic drivers of genetic patterns in three temperate reef species. Molecular Ecology, 19, 3708–3726. 10.1111/j.1365-294X.2010.04658.x 20723063

[ece36533-bib-0061] Shapiro, D. Y. (1983). On the possibility of kin groups in coral reef fishes. Ecology of Deep and Shallow Reefs, 1, 39–45.

[ece36533-bib-0062] Simons, A. M. (2004). Many wrongs: The advantage of group navigation. Trends in Ecology & Evolution, 19, 453–455. 10.1016/j.tree.2004.07.001 16701304

[ece36533-bib-0063] Staaterman, E. , & Paris, C. B. (2014). Modelling larval fish navigation: The way forward. ICES Journal of Marine Science, 71, 918–924. 10.1093/icesjms/fst103

[ece36533-bib-0064] Thorrold, S. R. , Jones, G. P. , Hellberg, M. E. , Burton, R. S. , Swearer, S. E. , Neigel, J. E. , … Warner, R. R. (2002). Quantifying larval retention and connectivity in marine populations with artificial and natural markers. Bulletin of Marine Science, 70, 291–308.

[ece36533-bib-0065] Toonen, R. J. , & Pawlik, J. R. (2001). Foundations of gregariousness: A dispersal polymorphism among the planktonic larvae. Evolution, 55, 2439–2454. 11831660 10.1111/j.0014-3820.2001.tb00759.x

[ece36533-bib-0066] Torney, C. J. , Berdahl, A. , & Couzin, I. D. (2011). Signalling and the evolution of cooperative foraging in dynamic environments. PLoS Computational Biology, 7, 1–10. 10.1371/journal.pcbi.1002194 PMC3178622 21966265

[ece36533-bib-0067] Torney, C. , Neufeld, Z. , & Couzin, I. D. (2009). Context‐dependent interaction leads to emergent search behavior in social aggregates. Proceedings of the National Academy of Sciences of the United States of America, 106, 22055–22060. 10.1073/pnas.0907929106 20018696 PMC2799714

[ece36533-bib-0068] Warner, R. R. , & Hughes, T. P. (1988). The population dynamics of reef fishes. Proceedings of the 6th International Coral Reef Symposium, 1, 149–155.

[ece36533-bib-0069] Watson, J. , Mitarai, S. , Siegel, D. , Caselle, J. E. , Dong, C. , & McWilliams, J. (2010). Realized and potential larval connectivity in the Southern California Bight. Marine Ecology Progress Series, 401, 31–48. 10.3354/meps08376

[ece36533-bib-0070] Weersing, K. , & Toonen, R. (2009). Population genetics, larval dispersal, and connectivity in marine systems. Marine Ecology Progress Series, 393, 1–12. 10.3354/meps08287

[ece36533-bib-0071] Werner, F. E. , Page, F. H. , Lynch, D. R. , Loder, J. W. , Lough, R. G. , Perry, R. I. , … Sinclair, M. M. (1993). Influences of mean advection and simple behavior on the distribution of cod and haddock early life stages on Georges Bank. Fishries Oceanography, 2, 43–64. 10.1111/j.1365-2419.1993.tb00120.x

[ece36533-bib-0072] Wong, M. Y. L. , Fauvelot, C. , Planes, S. , & Buston, P. M. (2012). Discrete and continuous reproductive tactics in a hermaphroditic society. Animal Behaviour, 84(4), 897–906.

