# Supplementary material for: Travel with your kin ship! Insights from genetic sibship among settlers of a coral damselfish
Source: Ecol Evol. 2020 Jul 14;10(15):8265–78. doi: 10.1002/ece3.6533 (PMC7417242; doi:10.1002/ece3.6533)
Supplement: Supplementary file 1 — Supplementary Material [file ECE3-10-8265-s001.docx]

**Supplementary Information**

**Appendix S1:** Biology and ecology of *Dascyllus aruanus* as reference for inferences of recruitment in *D. abudafur*

*Dascyllus aruanus* is a small coral-dwelling damselfish, widely distributed throughout the Indo-Pacific. Its individuals use the entire coral colony equally as their home (Sale, 1970), though there is a strict protogynous haremic size-dominated hierarchy, in which the largest specimen is the reproductive male and is usually accompanied by three reproductive females and several other smaller juveniles, unless the coral colony is much bigger in size (increasing the number of mature males) or inhabited by less individuals (resulting in less mature females and juveniles; Fricke & Holzberg, 1974; Coates, 1982; Shpigel & Fishelson, 1986; Schwarz & Smith, 1990). A similar structure has also been observed among *D. abudafur* in the Red Sea (personal observation). The reproductive females of *D. aruanus* lay egg clutches of 100-1500 eggs (average size of 830 eggs per three females, Wong et al., 2012) at the bottom of the coral colony, which get then fertilized by the mature male, hatch after two to three days (Mizushima et al., 2000), and will complete their pelagic larval duration (PLD) in an average of about three weeks (average of 21.1 d, across all published studies; Luiz et al., 2013). Similar to *D. aruanus,* the PLD of *D. abudafur* varies with location with an average 23.2 d in the Red Sea (Robitzch et al., 2016). The growth rate of *D. aruanus* is of ~ 5.08 mm per month during the first year, putatively reaching maturity at a size of ~ 38 mm (but probably at a smaller size for the *D. abudafur* of the Red Sea, personal observation) and a size of ~ 61mm at the end of its first year (Pillai et al., 1985a). Breeding peaks have been reported from April to January for Minicoy Atoll in the northern Indian Ocean (Pillai et al., 1985a), and spawning peaks from June to September in Japan. In Japan, *D. aruanus* follows a semilunar spawning cycle usually two to four days directly before or around new and full moon, and during this period 33% of the populations’ females spawn in intervals of approximately two weeks (13-59 days; mode=14) one to six times (mode=4) per spawning season (Mizushima et al., 2000). *Dascyllus aruanus* has typically restricted mobility due to predation pressure and habitat patchiness (Fricke, 1980; Shpigel & Fishelson, 1986) but exhibits opportunistic behavior when it comes to feeding (Pillai et al., 1985a, 1985b), which was recently also documented among *D. abudafur* (Robitzch et al., 2019).

**Appendix S2:** Double digest restriction associated DNA (ddRAD) library preparation for *Dascyllus abudafur*

After high quality DNA extraction from the 171 individuals of the recruiting cohort sample (RCS) of *Dascyllus abudafur*, three individuals were excluded from the double digest restriction associated DNA (ddRAD) library preparation, because they yielded too low DNA concentrations (<10 ng/μl). Of the remaining 224 individuals (RCS + adult population sample (APS)), 500 ng of genomic DNA was digested at 37 °C for three hours using the restriction enzymes *SphI* and *MluCI* (NEB), followed by a ligation step, where each sample was assigned to one of sixteen unique adaptors. Pools of sixteen individuals were combined and run on a 1.5% agarose gel, from which fragments of ~400 base pair (bp) were manually excised and purified using a Zymoclean Gel DNA Recovery Kit. Each pool was then amplified adding a unique indexing primer for each pool according to the standard Illumina multiplexed sequencing protocol in a 50 μl PCR reaction containing 25 μl Kapa Hifi Hotstart Ready Mix Taq, 20 μl of pooled library DNA, 2.5 μl of the universal Illumina PCR primer, and an additional 2.5 μl of one of 12 unique indexing primers, for each pool. Amplifications were carried out in an Eppendorf 94-well vapo.protect Mastercycler Pro System (Fisher Scientific) using the following protocol: initial step at 95 °C for 3 min, followed by ten cycles of 98 °C for 20 s, 60 °C for 30 s, and 72 °C for 30 s, and a final step at 72 °C for 5 min. DNA libraries were then quantified using the High Sensitivity DNA Analysis Kit in a 2100 Bioanalyzer (Agilent Technologies) and by running a qPCR in an ABI 7900HT fast real-time PCR system (Thermo Fisher Scientific) using the KAPA Library Quantification Kits (Kapa Biosystems). One last time, the length in bp and quality of the library fragments was measured in the 2200 TapeStation (Agilent Technologies) using the High Sensitivity D1000 ScreenTape Kit. Pools were subsequently combined in equimolar concentration to form a single genomic library. In total, two libraries were created and run twice on two separated lanes of a HiSeq 2000 Illumina sequencer (four lanes total, two lanes for each of the two libraries containing 112 individuals each; single end reads, 1 x 101 bp; v3 reagents) to increase the coverage per locus.

**Appendix S3:** Details on the STACKS (v.1.42) pipeline used for processing ddRAD data for *Dascyllus abudafur*

Parameter for the *de-novo* assembly were: Minimum read depth to create a stack (-m) = 6, number of mismatches allowed between loci within individuals (-M) = 3, number of mismatches allowed between loci within catalog (-n) = 2, and the option to remove or break up highly repetitive RAD-Tags in the *ustacks* program (-t).

Within *de-novo* mapping, the *population* component of STACKS was used for the following filtering: First, only those loci present in at least 95% of individuals were retained (-r) = 0.95. Second, all loci with minor allele frequencies lower than 0.05 were removed to reduce the number of false polymorphic loci due to sequencing error (--min_maf) = 0.05. Third, the maximum observed heterozygosity required to process a nucleotide site at a locus was set to 60% to filter loci out of Hardy-Weinberg-Equilibrium (HWE) (--max_obs_het) = 0.6. As a last step, the log likelihood (lnl) ratio of all loci was calculated using *rxstacks* and after looking at its distribution a log likelihood threshold of (--lnl_lim) = -5 was used to filter loci with lnl values below -5. The *write_random_snp* option produced a vcf file with only one single randomly chosen nucleotide polymorphism (SNP) per stack.

**Appendix S4:** Details on the calculation of the size of two putative total recruiting cohort population (TRCP) of *Dascyllus abudafur*

In order to infer an initial size for a theoretical total recruiting cohort (TRC) for *Dascyllus abudafur* from the Red Sea, Al Karrah (AKA) reef, we used this formula: TRCP_r_ = R_Cd_ * C_A_ * F_R%_ * A_r_. The exact values for each factor can be found in Table 2 and are calculated step by step in the following. For (a) R_Cd_ (= E * H * Hd_%_ * S): the percentage that hatched per day is (Hd_%_) = 0.5 (as larvae tend to hatch after two to three days (Mizushima et al., 2000); the survival rate of the PLD (S) = from 0.01 to 0.05 (in agreement with Giacomo Bernardi and Ricardo Beldade); so that the minimum R_Cdmin_ = 830 * 0.5 * 0.5 * 0.01 = 2, and the maximum R_Cdmax_ = 830 * 0.5 * 0.5 * 0.05 = 10, and thus the final averaged R_Cd_ = (2+10)/2 = 6. For (b) C_A_ the average number of *D. abudafur* colonies (Da) per 50 m * 2 m transect = 10.23, so that C_A_= 10.23/0.1km^2^ = 102/km^2^. For (c) F_R%_, we chose average values reported by Mizushima et al. (2000), so that F_R%_= 0.33. The presence of at least 3 reproductive females per colony (i.e., F_R%_= 0.33) in the Red Sea, was cross-checked by calculating the average number of individuals per colony, which was 17 individuals for AKA, and 7 for the entire Red Sea data. Both values are above four and thus large enough to contain at least the assumed average of three reproductive females per colony (> F_R%_). For (d) A_r_, the trajectory of potential habitat for *D. abudafur* (L) measured for a radius of 25 km and 50 km was L_r25_= 160 km and L_r50_= 387 km, respectively; and the average “width” of *D. abudafur* habitat (W) was set to be W = 1.2 km, following averages of bathymetry measurements from central Red Sea reefs (by VR, data not shown), and using a depth range from 1 m to 19 m as suitable (bathymetry by Maha Khalil); so that A_r25_  = 160 km * 1.2 km = 192 km^2^; and A_r50_  = 192 km^2^ + 227 km * 1.2 km = 464.4 km^2^.

Therefore, TRCP_r25_ = R_Cd_ * C_A_ * F_R%_ * A_r25_ = 19445; and TRCP_r50_ = R_Cd_ * C_A_ * F_R%_ * A_r50_ = 47033, at AKA.

**References**

Coates, D. (1982) Some observations on the sexuality of humbug damselfish, *Dascyllus aruanus* (Pisces, Pomacentridae) in the field. *Zeitschrift für Tierpsychologie*, **59**, 7–18.

Fricke, H.W. (1980) Control of different mating systems in a coral reef fish by one environmental factor. *Animal Behaviour*, **28**, 561–569.

Fricke, H.W. & Holzberg, S. (1974) Social units and hermaphroditism in a pomacentrid fish. *Naturwissenschaftern*, **61**, 367–368.

Luiz, O.J., Allen, A.P., Robertson, D.R., Floeter, S.R., Kulbicki, M., Vigliola, L., Becheler, R., & Madin, J.S. (2013) Adult and larval traits as determinants of geographic range size among tropical reef fishes. *PNAS*, **110**, 16498–16502.

Mizushima, N., Nakashima, Y., & Kuwamura, T. (2000) Semilunar spawning cycle of the humbug damselfish *Dascyllus aruanus*. *Journal of Ethology*, **18**, 105–108.

Pillai, C.S.G., Mohan, M., & Koya, K.K.K. (1985a) Ecology and biology of the white tailed humbug *Dascyllus aruanus* (Pomacentridae, Pisces) from Minicoy Atoll. *Journal of the Marine Biological Association of India*, **27**, 113–123.

Pillai, C.S.G., Mohan, M., & Kunhikoya, K.K. (1985b) A critique on the relationship of surface area of live coral with total number of fishes as well as the biomass of fish in a coexisting system of *Chromis caeruleus* and *Dascyllus aruanus* (Pomacentridae) at Minicoy Atoll. *Marine Biological Association of India*, **27**, 1–8.

Robitzch, V., Chen, C.T., Sturaro, N., Lepoint, G., Berumen, M.L., & Frédérich, B. (2019) “Homemade”: The phenotypic diversity of coral reef damselfish populations is driven by the local environment. *Biological Journal of the Linnean Society*, **127**, 361–376.

Robitzch, V., Lozano-Cortés, D., Kandler, N.M., Salas, E., & Berumen, M.L. (2016) Productivity and sea surface temperature are correlated with the pelagic larval duration of damselfishes in the Red Sea. *Marine Pollution Bulletin*, **105**, 566–574.

Sale, P. (1970) Behaviour of the humbug fish. *Australian Natural History*, **16**, 362–366.

Schwarz, A. & Smith, L. (1990) Sex change in the damselfish *Dascyllus reticulatus* (Richardson)(Perciformes: Pomacentridae). *Bulletin of Marine Science*, **46**, 790–798.

Shpigel, M. & Fishelson, L. (1986) Behavior and physiology of coexistence in two species of *Dascyllus* (Pomacentridae, Teleostei). *Environmental Biology of Fishes*, **17**, 253–265.

Wong, M.Y.L.Y.L., Fauvelot, C., Planes, S., & Buston, P.M. (2012) Discrete and continuous reproductive tactics in a hermaphroditic society. *Animal Behaviour*, **84**, 897–906.
